# Efficacy and Durability of Immune Response After Receipt of Hepatitis A Vaccine in People With Human Immunodeficiency Virus

**DOI:** 10.1093/ofid/ofaf143

**Published:** 2025-04-11

**Authors:** Bahaa Kazzi, Amal Naji, Serena Maria Dib, Lana Khalil, Sonia Tandon Wimalasana, Diane Saint-Victor, Ighovwerha Ofotokun, Nadine Rouphael

**Affiliations:** The Hope Clinic of the Emory Vaccine Center, Division of Infectious Diseases, Department of Medicine, Emory University, Decatur, Georgia, USA; The Hope Clinic of the Emory Vaccine Center, Division of Infectious Diseases, Department of Medicine, Emory University, Decatur, Georgia, USA; The Hope Clinic of the Emory Vaccine Center, Division of Infectious Diseases, Department of Medicine, Emory University, Decatur, Georgia, USA; The Hope Clinic of the Emory Vaccine Center, Division of Infectious Diseases, Department of Medicine, Emory University, Decatur, Georgia, USA; The Hope Clinic of the Emory Vaccine Center, Division of Infectious Diseases, Department of Medicine, Emory University, Decatur, Georgia, USA; Children's Healthcare of Atlanta, Division of Infectious Diseases, Department of Pediatrics, Emory University School of Medicine, Atlanta, Georgia, USA; The Hope Clinic of the Emory Vaccine Center, Division of Infectious Diseases, Department of Medicine, Emory University, Decatur, Georgia, USA; The Hope Clinic of the Emory Vaccine Center, Division of Infectious Diseases, Department of Medicine, Emory University, Decatur, Georgia, USA

**Keywords:** hepatitis A vaccines, hepatitis A virus, HIV, immunogenicity, vaccine durability

## Abstract

Hepatitis A virus (HAV) infection is a serious health concern among people with human immunodeficiency virus (HIV). Coinfection with HAV and HIV is linked to increased hepatitis A viral load, elevated HIV RNA, and potential disruption of HIV treatment caused by liver dysfunction. Three vaccines for the prevention of HAV are currently approved for usage in the United States: 2 monovalent inactivated vaccines (hepatitis A vaccine, inactivated [GSK] and hepatitis A vaccine, inactivated [Merck]) and 1 hepatitis A (inactivated) and hepatitis B (recombinant) vaccine (GSK). Among people with HIV (PWH), seroconversion rates and antibody titers to HAV vaccines tend to be lower and less persistent than in immunocompetent individuals, with a notable difference among PWH with a lower CD4 cell count. We highlight in this review the potential need for serologic monitoring and revaccination strategies that would optimize lifelong protection against HAV in PWH.

## Teaser

Hepatitis A vaccination in people with HIV leads to lower seroconversion rates and less durable antibody responses compared to immunocompetent individuals. We highlight in this review the potential need for serologic monitoring and revaccination strategies that would optimize lifelong protection.

Hepatitis A virus (HAV) can lead to fulminant hepatitis in less than 1% of cases [1]. The virus can be transmitted through the fecal-oral route, such as the consumption of contaminated food or water, household or sexual contact, exposure in daycare centers and residential institutions, and intravenous drug use. HAV causes self-limited disease, typically characterized by fatigue, jaundice, fever, nausea, vomiting, and abdominal pain [2]. HAV tends to be less severe in healthy individuals than in those who are immunocompromised.

Despite extensive public health efforts, HAV outbreaks persist worldwide. In 2019, the World Health Organization (WHO) documented a growing global burden of HAV, with 159 million cases of acute infection resulting in 39 000 deaths. The rising incidence of HAV is thought to be secondary to inadequate sanitary conditions and hygiene practices [2]. In the United States (US), the number of new HAV cases reported to the Centers for Disease Control and Prevention (CDC) was 2265 in 2022 [3]. Individuals between 30 and 39 years of age exhibit the highest rate of HAV compared to other age groups [3]. Moreover, vulnerable populations, including individuals experiencing homelessness, people who use illicit drugs, men who have sex with men (MSM), and people with human immunodeficiency virus (HIV), experience particularly high incidence rates [4]. Among people with HIV (PWH), older age and the injection of drugs are associated with higher seropositivity [5]. HAV infection in PWH is associated with adverse outcomes, including a higher HAV load at the onset of symptoms, a prolongation of HAV viremia and fecal shedding, and delayed resolution of hepatitis [6–9]. HAV and HIV coinfection may increase the HIV viral load (VL) and the risk of HIV transmission, although some studies found no evidence of this association [8–10]. As there is no antiviral therapy against HAV, its prevention through vaccination is essential.

In this review, we aim to summarize current knowledge on HAV vaccine efficacy and immunogenicity in PWH, with a focus on the magnitude of the immune response, seroconversion rates, and durability, as well as possible strategies to enhance these outcomes.

## US FOOD AND DRUG ADMINISTRATION–APPROVED HAV VACCINES AND NATIONAL GUIDELINES FOR PWH

Hepatitis A vaccine, inactivated (GlaxoSmithKline Biologicals [GSK], Rixensart, Belgium) and hepatitis A vaccine, inactivated (Merck Sharp & Dohme Corp, Rahway, New Jersey) are the 2 monovalent vaccines currently approved for usage in the US [11, 12]. Hepatitis A vaccine, inactivated (GSK) is administered on a 0 and 6–12 months schedule while hepatitis A vaccine, inactivated (Merck) is administered on a 0 and 6–18 months schedule. Hepatitis A (inactivated) and hepatitis B (recombinant) vaccine (GSK) is also US Food and Drug Administration approved and is given as 3 doses at 0, 1, and 6 months [13].

The CDC recommends that all PWH aged 1 year or older be immunized and assessed for an antibody response at least 1 month after completion of the primary series [4]. In the absence of an antibody response (ie, ≥10 mIU/mL), CDC advises revaccinating the individual, particularly among PWH who demonstrate an improvement in immune status (eg, increased CD4 cell count and decreased HIV VL). At least 1 month after completion of the revaccination series, antibody levels should be checked. If the response to revaccination is still not adequate (ie, <10 mIU/mL), additional vaccination is not recommended. However, the patient should be counseled on ways to prevent HAV infection.

The updated European AIDS Clinical Society (EACS) guidelines published in 2023 recommends vaccination for PWH who are seronegative. EACS also recommends repeating vaccinations that were administered when the CD4 cell count was <200 cells/μL (or during unsuppressed HIV viremia) once the individual has achieved immune reconstitution [14]. In this scenario, EACS also advises against schedules with shorter interval between doses and recommends assessing for seroconversion. Table 1 provides a summary of CDC, EACS, British HIV Association, and WHO HAV vaccination guidelines among PWH.

**Table 1.** Hepatitis A Virus Vaccine Recommendations for People With Human Immunodeficiency Virus

Recommendation	CDC, 2023 [4, 15]	EACS, 2023 [14]	BHIVA, 2015 [16]	WHO, 2022 [2]
Recommended age for vaccination	≥12 mo	12–23 mo	Nonimmune, at-risk	≥12 mo
Catch-up vaccination	Not specified	2–18 y	Not specified	Not specified
Number of doses	2 doses (at 0 and 6–12 mo for hepatitis A vaccine, inactivated [GSK] and 0 and 6–18 mo for hepatitis A vaccine, inactivated [Merck])^a^	2 doses	2 doses (at 0 and 6 mo for PWH with a CD4 cell count >350 cells/μL) or 3 doses (at 0, 1, and 6 mo for those with a cell count <350 cells/μL)	2 doses
Available vaccines	Hepatitis A vaccine, inactivated (GSK)^b^			
	Hepatitis A vaccine, inactivated (Merck)^b^			
	Hepatitis A (inactivated) and hepatitis B (recombinant) vaccine (GSK)^c,d^			
CD4 count and VL at time of vaccination	Not specified	CD4 count ≥200 cells/μL or ≥15% or suppressed viremia	3 doses if CD4 count <350 cells/mL	Not specified
Booster recommendations	No booster	No booster	For PWH with continued risk of exposure, administer a booster dose every 10 y	No booster
Repeat vaccination	In the absence of antibody response (ie, <10 mIU/mL)	After immune reconstitution if primary series was completed when CD4 <200 cells/μL or during unsuppressed HIV viremia	No repeat vaccination	No repeat vaccination

Abbreviations: BHIVA, British HIV Association; CDC, Centers for Disease Control and Prevention; EACS, European AIDS Clinical Society; GSK, GlaxoSmithKline; HIV, human immunodeficiency virus; PWH, people with human immunodeficiency virus; VL, viral load; WHO, World Health Organization.

^a^Or 3 doses (at 0, 1, and 6 months) when combination vaccine is used.

^b^Single-antigen vaccine.

^c^Combination vaccine.

^d^Not preferred due to reduced immunogenicity (BHIVA).

HAV vaccines have proven to be effective in reducing the risk of infection in immunocompetent individuals. The protective efficacy of hepatitis A vaccine, inactivated (GSK) was measured at 94% in a randomized controlled trial (RCT) in Thailand [17]. Furthermore, in a double-blind, placebo-controlled trial, the seroconversion rate of hepatitis A vaccine, inactivated (Merck) was measured at 100% in immunocompetent children (2 through 16 years of age) who were seronegative at baseline [18]. A comparative clinical trial between hepatitis A (inactivated) and hepatitis B (recombinant) vaccine (GSK) and hepatitis A vaccine, inactivated (GSK) also indicated equal seroconversion rates between vaccine recipients [19].

HAV vaccination campaigns in PWH were found to be efficient at curbing infection rates [20–22]. A modeling study on the effectiveness of HAV vaccines among PWH during the 2015–2017 hepatitis A outbreak in Taiwan indicated that vaccination greatly limited the number of infection [21]. Specifically, 1352 HAV cases were recorded while an estimated 7153 cases would have occurred in the absence of vaccination. This equates to an 80.7% decrease in the number of infections. This reduction demonstrates the substantial effectiveness of vaccination campaigns in curbing the spread of hepatitis A in this population.

## IMMUNOGENICITY AND SAFETY OF HAV VACCINES IN PWH

In a double-blind trial, Wallace et al assessed the immunogenicity of hepatitis A vaccine, inactivated (Merck) between 90 people without HIV (PWoH) and 60 PWH. Thirty separate PWH received a placebo [23]. At week 28, 4 weeks after the second vaccination, the seropositivity rate (defined as antibody titers ≥10 mIU/mL) among PWoH was 100%, versus 94% among PWH. Some studies reported considerably lower seroconversion rates following HAV vaccination in PWH [24–29]. A Bayesian meta-analysis of 8 studies (with a combined 458 patients) indicated that seroconversion rates in PWH could be closer to 64% (by intent-to-treat [ITT] analysis) [24]. Table 2 summarizes the results from studies assessing the immunogenicity of HAV vaccines in PWH.

**Table 2.** Summary of Evidence From Studies Assessing the Immunogenicity of Hepatitis A Virus Vaccines in People With Human Immunodeficiency Virus

Study	Population	Sample Size	Relevant Methodology Information	Vaccine Received; Number and Timeline of Doses Administered	Study Design	CD4 Count (Cells/μL)	VL (Copies/mL)	Duration of Follow-up	Seropositivity in Vaccinated Group at the End of Follow-up	Other Important Findings
Wallace et al (2004) [23]	Adults (mean age of PWH receiving hepatitis A vaccine, inactivated [Merck]: 32.6 y; mean age of PWH receiving placebo: 33.5 y; mean age of PWoH receiving hepatitis A vaccine, inactivated [Merck]: 33.6 y)	180 (60 PWH receiving hepatitis A vaccine, inactivated [Merck]; 30 PWH receiving placebo; 90 PWoH receiving hepatitis A vaccine, inactivated [Merck])	Stratification by CD4 cell count with a cutoff of 300 cells/μL	Hepatitis A vaccine, inactivated (Merck) or placebo; 2 doses (weeks 0 and 24)	Double-blind	PWH receiving hepatitis A vaccine, inactivated (Merck): 457.5 (mean) PWH receiving placebo: 493.6 (mean)	PWH receiving hepatitis A vaccine, inactivated (Merck): 0.33 × 10^5^ (mean); PWH receiving placebo: 0.16 × 10^5^ (mean)	52 wk	PWH: 90% PWoH: 100%	Participants with a CD4 count ≥300 cells/μL had a seroconversion rate equal to that of PWoH at week 52 (100%; [95% CI, 87%–100%]) compared to 87% (95% CI, 66%–97%) in PWH with a CD4 <300 cells/μL.
Shire et al (2005) [24]	Participants from 8 studies	458	A Bayesian hierarchical random-effects model was used in order to estimate the overall response to vaccine. Heterogeneity between individual studies was significant.	Varies between studies	Bayesian meta-analysis	…	…	…	Observed response rates varied from 50% to 95%. The ITT analysis estimated a response rate of 64% (95% CI, 52%–75%). In the PP analysis, the response rate was 71% (95% CI, 60%–80%).	Up to one-half of PWH may be nonresponders.
Weinberg et al (2006) [30]	CWH with evidence of immunologic reconstitution on HAART (median age at enrollment: 9 y)	235	At baseline, 152 participants were HAV seronegative, 48 were HAV seropositive, and 35 did not have their anti-HAV antibody titers measured.	Hepatitis A vaccine, inactivated (GSK); 2 doses (weeks 0 and 24). A subset of participants received a third dose at week 104.	Prospective study	856 (median)	483 (median)	104 wk	90%	Eight weeks after the administration of the third dose, 97% had protective antibody titers. The proportion of participants with antibody titers ≥250 mIU/mL was significantly higher after the third dose than the second dose.
Launay et al (2008) [31]	PWH (mean age of 3-dose group: 38.7 y; mean age of 2-dose group: 38.9 y)	95 (3-dose: 46; 2-dose: 49)	…	Hepatitis A vaccine, inactivated (GSK) (2-dose: weeks 0 and 24; 3-dose: weeks 0, 4, and 24)	RCT	3-dose: 351 (median) 2-dose: 355 (median)	3-dose: <50 (median) 2-dose: <50 (median)	72 wk	ITT analysis: 3-dose: 78.3%; 2-dose: 61.2% (*P* = .007) Observed analysis: 3-dose: 85.7%; 2-dose: 69.8% (*P* = .008)	The 3-dose group had significantly higher antibody titers than the 2-dose group at week 28 (323 vs 158 mIU/mL; *P* = .03) and week 72 (132 vs 67 mIU/mL; *P* = .05).
Tseng et al (2013) [32]	Adults (mean age of 2-dose PWH: 31.0 y; mean age of 3-dose PWH: 29.8 y; mean age of 2-dose PWoH: 26.4 y)	582 (2-dose PWH: 140; 3-dose PWH: 225; 2-dose PWoH: 217)	MSM	Hepatitis A vaccine, inactivated (GSK) (2-dose: weeks 0 and 24; 3-dose: weeks 0, 4, and 24)	Prospective cohort study	2-dose PWH: 538 (mean) 3-dose PWH: 452 (mean)	2-dose: 2.5 log_10_ copies/mL (mean) 3-dose: 3.0 log_10_ copies/mL (mean)	72 wk	2-dose: 86.6% 3-dose: 86.9%	The titers among the 3-dose cohort of PWH was significantly higher than in the 2-dose cohort of PWH at week 48 (2.29 ± 0.73 vs 1.94 ± 0.66 log_10_ mIU/mL, *P* < .01) and week 72 (2.08 ± 0.68 vs 1.78 ± 0.56 log_10_ mIU/mL, *P* < .01).
Lin et al (2018) [28]	PWH (mean age of hepatitis A vaccine, inactivated [GSK]- hepatitis A vaccine, inactivated [Merck] group: 35 y; mean age of hepatitis A vaccine, inactivated [Merck] group: 34 y)	946 (hepatitis A vaccine, inactivated [GSK]- hepatitis A vaccine, inactivated [Merck]: 395; hepatitis A vaccine, inactivated [Merck]: 551)	Due to a shortage of hepatitis A vaccine, inactivated (GSK), individuals included received either hepatitis A vaccine, inactivated (GSK) followed by hepatitis A vaccine, inactivated (Merck), or 2 doses of hepatitis A vaccine, inactivated (Merck).	Hepatitis A vaccine, inactivated (GSK)-hepatitis A vaccine, inactivated (Merck) or 2 doses of hepatitis A vaccine, inactivated (Merck)	Retrospective study	Hepatitis A vaccine, inactivated (GSK)–hepatitis A vaccine, inactivated (Merck): 571 (median) Hepatitis A vaccine, inactivated (Merck): 577 (median)	Hepatitis A vaccine, inactivated (GSK)–hepatitis A vaccine, inactivated (Merck): <20 (median) Hepatitis A vaccine, inactivated (Merck): <20 (median)	48 wk	Hepatitis A vaccine, inactivated (GSK)–hepatitis A vaccine, inactivated (Merck): 94.7% Hepatitis A vaccine, inactivated (Merck): 94.4%	The seroconversion rate after the first dose of hepatitis A vaccine, inactivated (Merck) was significantly higher than after hepatitis A vaccine, inactivated (GSK) (53.0% vs 32.4%).
Neukam et al (2019) [33]	PWH, mainly MSM (86.2%) (median age of 2-dose group: 26 y; median age of the 1-dose group: 38 y)	522 (2-dose: 353; 1-dose: 179)	Only qualitative anti-HAV analysis was conducted.	Hepatitis A vaccine, inactivated (GSK) (1 or 2 doses given 6 mo apart)	Retrospective study	2-dose: 595 (median) 1-dose: 676 (median)	Not specified	12 mo	2-dose: 88.3% 1-dose: 83.2%	A response rate >95% was achieved when the plasma HIV RNA load was undetectable and when the CD4 cell count was ≥350 cells/μL.
Noel et al (2021) [34]	PWH (median age: 49.4 y)	73	…	Hepatitis A vaccine, inactivated (Merck) (1 dose)	Retrospective study	658 (median)	Not specified	>30 d after vaccine administration	59.7% (106 d after injection in median)	Responders had a significantly higher CD4/CD8 ratio than nonresponders.
Tsai et al (2022) [35]	Adult men with HIV (mean age of 1-dose group: 33.26 y; mean age of 2-dose group: 31.99 y)	180 (90 participants received 1 dose of HAV vaccine; 90 age-matched controls received 2-dose)	There were more participants who injected drugs (22.22% vs 1.11%, *P* < .0001), or who were coinfected with hepatitis C (28.89% vs 5.56%, *P* < .0001), or who had a history of syphilis infection (56.67% vs 30%, *P* = .0003) in the 1-dose than in the 2-dose group.	Hepatitis A vaccine, inactivated (Merck) (1 or 2 doses 6 mo apart)	1:1 single-center retrospective case-control study	1-dose: 610.09 (mean) 2-dose: 648.86 (mean)	1-dose: 4076.48 (mean) 2-dose: 3118.55 (mean)	12 mo	1-dose: 56.67% 2-dose: 97.78%	Within the 1-dose group, people with HBV or HCV coinfections were less likely to achieve seropositivity. Those who had a higher CD4 cell count at baseline and at the 1-y follow-up had a better response to the vaccine.
Lin et al (2018) [22]	PWH (median age of vaccinated group: 35 y; median age of unvaccinated group: 33 y)	1533 (vaccinated: 1001; unvaccinated: 532)	The primary endpoints were seroconversion rates in the vaccinated group and the incidence rate of acute HAV infection.	Hepatitis A vaccine, inactivated (GSK) or hepatitis A vaccine, inactivated (Merck) (at least 6 mo apart). Due to a shortage, hepatitis A vaccine, inactivated (GSK) was substituted with hepatitis A vaccine, inactivated (Merck). By the end of the study, 965/1001 participants in the vaccinated group received 2 doses of HAV vaccine (96.4%).	Observational study	Vaccinated: 554 (median) Unvaccinated: 540 (median)	Vaccinated: <20 copies/mL (median) Unvaccinated: <20 copies/mL (median)	48 wk	ITT analysis: 55.4% ITT analysis using LOCF approach: 88.4% PP analysis: 94.5%	The incidence rate of acute HAV infection 3.7 in the vaccinated group and 99.3 per 1000 person-years in the unvaccinated group. This translates to a vaccine effectiveness of 96.3%.

Abbreviations: CI, confidence interval; CWH, children with human immunodeficiency virus; GSK, GlaxoSmithKline; HAART, highly active antiretroviral therapy; HAV, hepatitis A virus; HBV, hepatitis B virus; HCV, hepatitis C virus; HIV, human immunodeficiency virus; ITT, intent-to-treat; LOCF, last-observation-carried-forward; MSM, men who have sex with men; PP, per-protocol; PWH, people with human immunodeficiency virus; PWoH, people without human immunodeficiency virus; RCT, randomized controlled trial; VL, viral load.

A clinical trial with children with HIV (CWH) who were receiving highly active antiretroviral therapy (HAART) evaluated the immunogenicity of an HAV booster vaccine dose [30]. CWH who were eligible to participate received the first dose at enrollment and the second dose 24 weeks later. A subset of participants also received a booster dose at week 104 or later. Two hundred thirty-five CWH with CD4 cell percentages ≥20% were enrolled in the study. Four weeks after the second dose, 97% of participants who were seronegative at baseline seroconverted, and the median antibody titer was 320 mIU/mL. The proportion of participants who mounted an antibody response ≥250 mIU/mL was significantly higher after the third dose than after the second vaccine dose (76% vs 53%; *P* = .0002). The median antibody titers were also significantly higher after the third dose than after the second dose (602 vs 287 mIU/mL; *P* < .0001). The significantly higher antibody titers and the increased proportion of participants achieving antibody levels ≥250 mIU/mL after the third dose underscore the enhancement in immune response magnitude provided by a booster dose among CWH. The authors of this study also assessed the immunologic factors associated with mounting a protective antibody response among CWH after a 2-dose regimen [30]. High CD4^+^ B-lymphocyte percentage, high CD19^+^ percentage, and undetectable HIV VL at the time of first vaccine were associated with antibody response similar to that of children without HIV historical controls; however, this effect was not seen after the second vaccine. High antibody titers were generated after a third dose, regardless of CD4 percentage (at baseline or week 24) or VL (at week 104), suggesting that effects of booster doses may be less affected by stage of HIV infection in CWH.

As far as vaccine efficacy, in a prospective study on the serologic response of HAV vaccines in PWH during the 2015 hepatitis A outbreak in Taiwan, 1001 participants were followed over time. Receipt of at least 1 dose of HAV vaccine resulted in seroconversion rates of 63.8% (638/1001) at week 28–36 and 55.4% at week 48 (555/1001) [22]. Additionally, the incidence rate of acute HAV infection was 3.7 and 99.3 per 100 000 person-years in the vaccinated and unvaccinated groups, respectively.

Studies on HAV vaccination in PWH reported that the vaccines are generally well-tolerated without safety concerns, with no impact on CD4 cell count or HIV VL [23, 36–38], as supported by a double-blind placebo RCT comparing the administration of 2 doses of hepatitis A vaccine, inactivated (Merck) in PWH and PWoH [23]. Similarly, a prospective study assessing the safety and immunogenicity of the administration of 2 doses of the hepatitis A vaccine, inactivated (GSK) in MSM with or without HIV [36] had consistent safety results, with local soreness at the injection site being the most common side effect. Mild systemic symptoms (such as headache, rash, nausea, lightheadedness, and myalgia) were reported in 33% of PWH and 15% of PWoH.

## COMPARISON BETWEEN 1-, 2-, AND 3-DOSE VACCINATION REGIMENS IN PWH

Multiple studies assessed whether a 3-dose vaccination series improves seroconversion and magnitude of immune response in PWH. The data suggest that while the addition of a third dose may or may not improve seroconversion, the magnitude of the immune response is significantly higher [31, 32, 39]. There is evidence suggesting that higher antibody titers following hepatitis A vaccination are associated with longer-lasting immunity similar to hepatitis B. A study found that PWH with higher initial antibody concentrations postvaccination tended to maintain seropositivity for a longer duration [40]. Specifically, the estimated duration of antibody persistence ranged from 21 to 27 years, depending on the vaccination schedule and the initial antibody response. However, it is important to note that the anti-HAV concentration needed to confer protection against hepatitis A is unknown; the 10 or 20 mIU/mL cut points used in the studies were the lower limit of detection of the assays.

In an RCT comparing 2-dose and 3-dose vaccination series in PWH, Launay et al indicated that a 3-dose schedule led to higher, albeit statistically insignificant, seroconversion rates than a 2-dose schedule and significantly higher HAV antibody titers [31]. Participants were randomized to receive 3 doses of hepatitis A vaccine, inactivated (GSK) at weeks 0, 6, and 24, (46/95 patients) or 2 doses 24 weeks apart (49/95 patients). At week 28, seroconversion (defined as an anti-HAV antibody ≥20 mIU/mL) occurred in 82.6% of participants in the 3-dose group and in 69.4% of patients of the 2-dose group (ITT analysis, *P* = .13). At week 28, anti-HAV geometric mean titers (GMTs) were 323 and 138 mIU/mL in the 3-dose and 2-dose groups, respectively (*P* = .03). In the 3-dose and 2-dose groups, anti-HAV antibody GMTs decreased to 132 and 66 mIU/mL (*P* = .05) at week 72. Additionally, of the participants who had protective anti-HAV antibody titers at week 28, 94% still had persisting protective titers at week 72. The significantly higher GMTs observed in the 3-dose group compared to the 2-dose group suggest that the intensified schedule may lead to longer-lasting immunity [38]. Similarly, in a prospective cohort study, Tseng et al compared the immunogenicity between 3-dose and 2-dose primary series of hepatitis A vaccine, inactivated (GSK) in MSM with HIV, as well as a 2-dose series in MSM without HIV [32]. There were significant differences between the cohorts of participants with HIV in terms of age (2-dose PWH: 31.0 years, 3-dose PWH: 29.8 years; *P* = .03), mean baseline CD4 cell count (2-dose PWH: 538 cells/µL, 3-dose PWH: 452 cells/µL; *P* = .01), and mean baseline VL (2-dose PWH: 2.5 log_10_ copies/mL, 3-dose PWH: 3.0 log_10_ copies/mL; *P* < .01). At week 48, seroconversion rates were 75.7%, 77.8%, and 88.5% in the PWH 2-dose group, PWH 3-dose group, and in PWoH. The geometric mean concentration (GMC) of anti-HAV antibodies was also significantly higher in the PWH 3-dose arm (2.29 ± 0.73 log_10_ mIU/mL) than in the PWH 2-dose arm (1.94 ± 0.66; *P* < .01), but still lower than among PWoH (2.49 ± 0.42; *P* < .01).

Data on the efficacy of single-dose HAV vaccination has been conflicting, with some studies indicating that seroconversion rates are lower among PWH who receive 1 dose of HAV vaccine than among PWH receiving 2 doses [34, 35]. In a retrospective study, PWH achieved a seroconversion rate of 59.7% 2–6 months later [34]. Similarly, in a single-center retrospective case-control study, seroconversion rate 1 year after vaccination was 56.67% in PWH who received 1 dose, versus 97.78% in PWH who received the standard 2 doses (*P* < .001) [35]. These results conflict with those of a retrospective study, which did not show a statistically significant difference between 1-dose and 2-dose vaccinations in PWH (1-dose group: 83.2%; 2-dose group: 88.2%; *P* = .107) [33]. However, the duration of follow-up for the study was relatively short and the study population had well-controlled HIV.

## PREDICTORS OF SEROCONVERSION TO HAV VACCINE IN PWH

Multiple studies suggest that CD4 cell count in PWH is a main predictor of seroconversion. When stratifying by CD4 cell count, Wallace et al found that both PWH with a CD4 cell count ≥300 cells/μL and PWoH achieved a seroconversion rate of 100%, while PWH with a CD4 cell count <300 cells/μL had a noticeably lower response rate of 87% at week 28. These results are similar to other studies, which also find a correlation between CD4 cell count and seroconversion rates in PWH [23, 41–47]. Lower HIV VLs at the time of HAV vaccination are also associated with greater seroconversion rates [30, 33, 42, 48, 49], although other studies present conflicting data [43, 44]. Additionally, CD4/CD8 ratio may be associated with immune response following HAV vaccination [41, 50].

In a nonrandomized clinical trial on the immunological parameters of HAV vaccination in PWH, 63 PWH who were receiving HAART and 50 HAART-naive PWH were administered 2 doses of monovalent inactivated HAV vaccine [46]. One month after the second dose, there were no differences in seroprotection (PWH receiving HAART: 78%, treatment-naive PWH: 76%; *P* = .826) and anti-HAV antibody GMTs (237 mIU/mL vs 158 mIU/mL; *P* = .068) between the 2 arms [46]. These results are possibly attributable to similar CD4 cell counts at baseline. Additionally, the study reported that patients with a CD4 cell count ≥500 cells/μL were 4.3 times more likely to respond to the vaccine than those with a CD4 cell count between 200 and 499 cells/μL (80% vs 71%, respectively; *P* = .005). The only factor associated with immune response was the CD4 cell count at vaccination.

In a multicenter study, Huang et al assessed the factors behind early seroreversion following 2 doses of HAV vaccine among PWH [51]. A total of 1256 PWH who were vaccinated during the HAV outbreak in Taiwan from 2015 to 2017 were followed. After a median follow-up of 611 days since the first HAV vaccination, 49 of 1256 patients (3.9%) had seroreverted. The seroreversion rate increased to 6.8% for participants who had a CD4 cell count <350 cells/μL. In a case-control study, patients with a higher weight (adjusted odds ratio [aOR], 1.703 [95% confidence interval {CI}, 1.292–2.323], per 10-kg increment) and HIV viremia at vaccination (aOR, 2.922 [95% CI, 1.067–7.924]) were more likely to serorevert. In contrast, positive seroresponse at 6 months after vaccination (aOR, 0.059 [95% CI, .020–.154]) and a higher CD4 cell count at vaccination (aOR, 0.837 [95% CI, .7004–.979], per 100-cell/μL increment) were inversely associated with early seroreversion in multivariable analyses.

## DURABILITY OF ANTIBODY REPONSE OF HAV VACCINATION IN PWH

When assessing the durability of HAV vaccination in PWoH, studies indicate that the HAV vaccine provides durable protection with reported persistence levels of seroprotective anti-HAV antibodies ranging up to 15–20 years in 95%–100% of vaccinees [52, 53]. In a 17-year follow-up following a 3-dose vaccination series of hepatitis A vaccine, inactivated (GSK), seroprotective antibody titers were reported in 87%–100% of participants [54]. One study estimated that seropositive anti-HAV antibody levels would still be present in ≥95% of vaccinees 30 years after vaccination and ≥90% 40 years later [55–57].

Among PWH who respond to HAV vaccines after completion of the series, seroconversion wanes more quickly, as shown in a meta-analysis on the long-term serological responses to vaccines in PWH. Data analyzed from 5 HAV vaccine studies in PWH who responded to vaccination reported that pooled percentages of seroprotection against HAV infection were 92% after 2 years and 82% after 5 years [25]. In a retrospective study on the long-term durability of HAV vaccinations in 130 PWH, 89% of participants achieved a detectable vaccination response [49]. Among initial responders, 90% remained seropositive at 3-year follow-up, and 85% after 6–10 years. Additionally, the anti-HAV antibody GMTs among PWH were 154, 111, and 64 mIU/mL at 1, 3, and 6–10 years, respectively, versus 1734, 687, and 684 mIU/mL among PWoH. Table 3 summarizes evidence of the studies reporting the durability of HAV vaccine immune response after >2 years since initial vaccination in PWH.

**Table 3.** Summary of Evidence From Studies Reporting the Durability of the Hepatitis A Virus Vaccine Seroresponse Since Initial Vaccination in People With Human Immunodeficiency Virus

Study	Population	Sample Size	Relevant Methodology Information	Vaccine Received; Number and Timeline of Doses Administered	Study Design	CD4 Count (Cells/μL)	VL (Copies/mL)	Follow-up	Seropositivity in Vaccinated Group at the End of Follow-up	Other Important Findings
Kerneis et al (2014) [25]	Adults or children with HIV	…	For each study, the decrease of seroprotection was modeled using a log binomial generalized linear model. One retrospective and 4 prospective studies were included.	Varies between studies	Meta-analysis	…	…	Seroprotection at 2–5 y after the last vaccine administration	2 y: 92% 5 y: 82%	…
Crum-Cianflone et al (2011) [49]	PWH (median age: 35 y)	130	Participants were further stratified by CD4 cell count (CD4 cell count < or ≥350 cells/μL) and VL (HIV RNA < or ≥1000 copies/mL).	Hepatitis A vaccine, inactivated (GSK) or hepatitis A vaccine, inactivated (Merck) (2 doses administered 6–18 mo apart)	Retrospective study	461 (median)	Not specified	6–10 y (median time: 8.2 y)	Among initial responders to the vaccine, 85% were still seropositive.	More robust GMCs were observed among participants with a CD4 cell count ≥350 cells/μL or a VL <1000 copies/mL. There were no statistically significant differences in seropositivity rate at the 6–10 y follow-up when participant were stratified by CD4 cell count or VL (although the sample size was small for later timepoints).
Lin et al (2023) [58]	PWH (median age: 34 y)	986	Seroconversion rates were assessed among PWH who seronconverted within 12 mo after the second dose.	Hepatitis A vaccine, inactivated (GSK) or hepatitis A vaccine, inactivated (Merck) (2 doses). 35.6% received hepatitis A vaccine, inactivated (GSK) followed by hepatitis A vaccine, inactivated (Merck) due to vaccine shortage.	Retrospective study	587 (median)	<20 (median)	60 mo	ITT with LOCF: 90.7% PP: 97.4%	In the multivariable analysis, seroreversion at month 60 was associated with a higher BMI, lowest-ever CD4 cell count, VL <200 copies/mL at vaccination, and receipt of hepatitis A vaccine, inactivated (Merck) as first dose of HAV vaccine. Seroprotection remained >90% among participants who underwent vaccination while on ART.
Fritzsche et al (2019) [41]	PWH (median age of mono-vaccine arm: 31 y; median age of HAV/HBV co-vaccine arm: 39 y)	131 (HAV mono-vaccine arm: 54 y; HAV/HBV co-vaccine arm: 77 y)	Patients with preexisting Hepatitis B immunity received hepatitis A vaccine, inactivated (Merck). Patients lacking immunity to HAV and HBV received hepatitis A (inactivated) and hepatitis B (recombinant) vaccine (GSK).	Hepatitis A vaccine, inactivated (Merck) (2 doses) or hepatitis A (inactivated) and hepatitis B (recombinant) vaccine (GSK) (3 doses)	Retrospective cross-sectional study	Mono-vaccine arm: 535 (median) Co-vaccine arm: 440 (median)	Mono-vaccine arm: 187 (median) Co-vaccine arm: 50 (median)	The median time between last vaccine and serological control: 865 d (782 d for mono-vaccine arm; 897 d for co-vaccine arm)	Mono-vaccine and co-vaccine arms: 80.2% Mono-vaccine arm: 81.5% Co-vaccine arm: 79.2%	In the mono-vaccine arm, a higher CD4 cell count, higher CD4/CD8 ratio, and shorter time from vaccine administration to serological control were associated with a better vaccine response. In the co-vaccine group, younger age and female sex were associated with better vaccine response.
Cheng et al (2017) [39]	PWH, all males (mean age of 2-dose primary responders: 31.0; mean age of 3-dose primary responders: 29.8)	2-dose primary responders: 110 3-dose primary responders: 185	Seroconversion rates were assessed among participants who seroconverted within 6 mo after the last vaccine dose and before month 18.	Hepatitis A vaccine, inactivated (GSK) (2 doses 6 mo apart or 3 doses according to a 0, 1, and 6 mo schedule)	Prospective cohort study	2-dose primary responders: 560 (mean) 3-dose primary responders: 470 (mean)	2-dose primary responders: 2.5 log_10_, copies/mL (mean) 3-dose primary responders: 2.9 log_10_, copies/mL (mean)	60 mo	ITT analysis: 2-dose group: 76% 3-dose group: 79% PP analysis: 2-dose group: 94% 3-dose group: 88%	There was not a statistically significant difference in seroprotection persistence among primary responders in the 3-dose and 2-dose groups (ITT and PP analyses; *P* > .05). GMCs of anti-HAV IgG were consistently higher in the 3-dose group throughout the 5 y in which participants were followed. In the multivariable analysis, a 3-dose regimen compared to a 2-dose regimen was independently associated with sustained seroprotection (OR, 3.36 [95% CI, 1.14–9.93]).
Jablonowska & Kuydowicz (2014) [47]	PWH (mean age: 30.7 y)	Vaccination was completed in 83 individuals at the time of publication with 49 participants available at follow-up.	…	Hepatitis A vaccine, inactivated (GSK) (2 doses 6 mo apart)	Prospective study	450.1 (mean)	Not specified	60 mo	Among participants who were seropositive 1 y after vaccination, 75.5% still had protective antibodies.	…
Vázquez-Rosales et al (2024) [59]	CWH (mean age: 12.6 y)	19	Blood samples were taken 28 d and 7 y after the second vaccine dose for antibody measurement.	Hepatitis A vaccine, inactivated (GSK) (2 doses 6 mo apart)	Cohort study	Not specified	Not specified	7 y	79%	Median antibody levels at the 7-year follow-up were 56 mIU/mL.

Abbreviations: ART, antiretroviral therapy; BMI, body mass index; CI, confidence interval; CWH, children with human immunodeficiency virus; GMC, geometric mean concentration; GSK, GlaxoSmithKline; HAV, hepatitis A virus; HBV, hepatitis B virus; HIV, human immunodeficiency virus; IgG, immunoglobulin G; ITT, intent-to-treat; LOCF, last-observation-carried-forward; OR, odds ratio; PP, per-protocol; PWH, people with human immunodeficiency virus; VL, viral load.

Similarly, in a retrospective study on the durability of HAV vaccines in PWH, Lin et al analyzed the immunogenicity of the hepatitis A inactivated vaccines in 986 PWH who achieved seroconversion within 12 months after vaccination [58]. Due to an early shortage of hepatitis A vaccine, inactivated (GSK), the majority of participants received a combination of hepatitis A vaccine, inactivated (GSK) and hepatitis A vaccine, inactivated (Merck). At the 5-year follow-up, 90.7% (894/986) of participants who seroconverted remained seropositive. These findings suggest that while long-term seroprevalence in PWH is still present, PWH tend to lose protection more quickly than PWoH.

Other studies reported lasting immunogenicity up to 7 years in PWH [39, 41, 47, 58–60]. A prospective study on the durability of HAV vaccination indicated that 79.5% (66/83) of participants had a vaccine response 1 month after immunization [47]. In the 1-year and 5-year follow-ups, respectively 81.9% (50/61) and 75.5% (37/49) of responders who were available for follow-up were still seropositive. Similarly, studies among CWH found that anti-HAV antibodies persisted at follow-up. Gouvêa et al estimated that anti-HAV antibody levels in a group of CWH were 113.0 mIU/mL, and 79.3% (23/29) maintained seropositivity at the 7-year follow-up [60]. A separate study indicated lower median antibody concentrations (80 mIU/mL) but persistent seroprotective titers in 80% of patients at the 7-year follow-up [59].

In a nonrandomized prospective study comparing the durability of immune responses between 2-dose and 3-dose vaccination series in PWH, Cheng et al assessed the antibody persistence of 18- to 40-year-old MSM with HIV [39]. The study cohort included 365 subjects, 110 of whom received a 2-dose series versus 185 who received a 3-dose series. There were significant differences in mean CD4 cell count (2-dose series: CD4 was 560 cells/μL; 3-dose series: 470 cells/μL; *P* < .001), mean plasma HIV RNA load (2.5 vs 2.9 log_10_ copies/mL; *P* = .01), combination antiretroviral therapy coverage (70.0% vs 58.9%; *P* = .06), and syphilis coinfection (24.7% vs 14.2%; *P* = .03) between the arms at baseline. For the 2-dose-vaccinated primary responders, 90% and 76% at 18 and 60 months, respectively, were seropositive in the ITT analysis; 87% and 79% of participants in the 3-dose cohort were seropositive for the same timepoints. Of note, GMCs of specific anti-HAV immunoglobulin G were consistently higher in the 3-dose group throughout the 5 years in which participants were followed.

## OTHER IMMUNOLOGIC ASSESSMENTS

The mechanism by which the HAV vaccine induces an effective immune response relies on the interplay of various immunological components. It produces an immune response that is dependent on T cells, specifically the naive CD4 T cells, and leads to the proliferation of T-helper 1 (Th1) response [46]. This cascade ultimately results in the generation of antibodies and the cytotoxic response of CD8 cells [46]. This immune response is expected to be diminished in PWH [41, 46, 48], underscoring the importance of reaching and maintaining HIV virologic and immunologic control through antiretroviral therapy (ART) to achieve optimal vaccine efficacy [48].

When looking at other elements of the immune system, no data were found regarding the cytokine response to the HAV vaccine in PWH. However, a significant rise in the levels of interleukin (IL)–6, interferon-γ, and tumor necrosis factor–α was noted in healthy participants after the administration of a single dose of HAV vaccine [61]. IL-2 and IL-10 levels were found to be higher in patients after the administration of the second dose of the vaccine [61].

## STRATEGIES TO OPTIMIZE IMMUNE RESPONSE OF HAV VACCINATION IN PWH

Several strategies could improve the immune response in PWH to HAV vaccines. First, the administration of booster doses could be needed to maintain long-term seroconversion rates. A study evaluating the immunogenicity of booster doses in immunocompetent individuals found that it elicited an anamnestic response, significantly increasing GMTs from 242 IU/L at baseline to 3832 IU/L at day 14 and 5282 IU/L 30 days after booster administration [62]. Revaccination strategies have also been assessed in PWH [63, 64]. One RCT evaluated 1-dose and 2-dose revaccination strategies in PWH who were anti-HAV antibodies negative after HAV vaccination [64]. They reported that both revaccination strategies led to similar seroconversion rates at week 48 (2 doses vs 1 dose, 80.2% vs 71.4%; *P* = .20), but that antibody titers were consistently higher in the 2-dose group. Another retrospective study compared seroconversion rates between PWH who were revaccinated after losing protective antibodies and those who received primary vaccination. Seroconversion rates at week 28—4 weeks after the second dose—were higher among the revaccinated group (98.7% vs 62.7%; *P* < .001) as well as at week 48 (98.7% vs 92.7%; *P* = .06) [63]. Within this context, vaccinating PWH with a low CD4 count with a primary series, followed by 1 or 2 booster doses after immune reconstitution, may maximize protection both in the short and long term.

Another potential strategy to improve immune response would be the implementation of a 3-dose primary series, instead of the currently recommended 2-dose series [31, 32, 39]. Studies on this topic reported that 3 doses of HAV vaccines in CWH and PWH led to higher antibody levels, though seroconversion rates did not differ based on the number of doses [30–32, 39].

Another approach is to defer vaccination until immune recovery. Most studies associate higher seroconversion rates and antibody levels with higher CD4 cell counts [23, 41–47] and suppressed HIV VL [30, 42, 48, 49]. However, this strategy is not widely adopted by national guidelines, especially if there is a high risk of acquiring HAV.

To our knowledge, higher-dose formulations and alternative routes of administration have not been assessed as potential ways to improve immune responses to vaccination. Serologic monitoring should also be taken into consideration.

## CONCLUSIONS

The life expectancy of PWH has increased dramatically [65–68]. However, it is currently unclear how often PWH should be tested for the presence of HAV protective antibody levels and how often HAV vaccine booster doses should be administered. Studies thus far have shown that while PWH respond to HAV vaccination, seroconversion rates and antibody levels are significantly lower than among immunocompetent individuals. Additionally, evidence data suggest that antibody levels among PWH wane far more quickly. Within this context, strategies to improve antibody responses and the durability of HAV immunity include the use of 3 vaccine doses within the primary series, the timing of HAV vaccination after immune reconstitution, and the early detection of nonresponders to primary series who will require additional doses. Strategies such as intradermal administration, higher doses, or adjuvanted formulations have been successfully used to enhance immune responses to hepatitis B and influenza vaccines. However, there is currently no clear role for these approaches in the context of the hepatitis A vaccine. This is because hepatitis A vaccination is capable of generating a robust immune response, even in immunocompromised individuals, following additional doses. Further research is needed to explore the optimal strategies for populations with unique immunologic challenges while considering the effectiveness of existing vaccination protocols.

## Notes

***Author contributions.*** Conceptualization: B. K., A. N., and N. R. Writing—original draft preparation: B. K. and A.N. Writing—review and editing: S. M. D., L. K., S. T. W., D. S.-V., I. O., and N. R. All authors have read and agreed to the published version of the manuscript.

***Financial support***. D. S.-V. is supported by the National Institutes of Health (grant number T32AI074492).
